# Comprehensive Analysis of Copy Number Variation, Nucleotide Mutation, and Transcription Level of PPAR Pathway-Related Genes in Endometrial Cancer

**DOI:** 10.1155/2022/5572258

**Published:** 2022-01-13

**Authors:** Minghui Tang, Jingyao Wang, Liangsheng Fan

**Affiliations:** ^1^Department of Obstetrics and Gynecology, The First Affiliated Hospital of Guangzhou Medical University, Guangzhou 510120, China; ^2^Affiliated Cancer Hospital and Institute, Guangzhou Medical University, Guangzhou 510000, China

## Abstract

Endometrial cancer is a common malignant tumor in gynecology, and the prognosis of advanced patients is dismal. Recently, many studies on the peroxisome proliferator-activated receptor pathway have elucidated its crucial involvement in endometrial cancer. Copy number variation (CNA) and nucleotide mutations often occur in tumor tissues, leading to abnormal protein expression and changes in protein structure. We analyzed the exon sequencing data of endometrial cancer patients in the TCGA database and found that somatic changes in PPAR pathway-related genes (PPAR-related-gene) often occur in UCEC patients. Patients with CNA or mutation changes in the exon region of the PPAR-related-gene usually have different prognostic outcomes. Furthermore, we found that the mRNA transcription and protein translation levels of PPAR-related-gene in UCEC are significantly different from that of adjacent tissues/normal uterus. The transcription level of some PPAR-related-gene (DBI, CPT1A, CYP27A1, and ME1) is significantly linked to the prognosis of UCEC patients. We further constructed a prognostic predicting tool called *PPAR Risk score*, a prognostic prediction tool that is a strong independent risk factor for the overall survival rate of UCEC patients. Comparing to the typical TNM classification system, this tool has higher prediction accuracy. We created a nomogram by combining *PPAR Risk score* with clinical characteristics of patients in order to increase prediction accuracy and promote clinical use. In summary, our study demonstrated that PPAR-related-gene in UCEC had significant alterations in CNA, nucleotide mutations, and mRNA transcription levels. These findings can provide a fresh perspective for postoperative survival prediction and individualized therapy of UCEC patients.

## 1. Introduction

One of the most prevalent malignancies in the female reproductive system is uterine corpus endometrial carcinoma (UCEC), and its incidence has been rising in the past few years [1]. Most UCEC patients have a better prognosis after hysterectomy and adjuvant therapy. However, for advanced-stage patients, the benefit rate is less than 50% in current treatment strategies [2]. Therefore, it is still necessary to explore the pathogenesis of UCEC and treatments for advanced patients.

Peroxisome proliferator-activated receptors (PPARs) are transcription factors of the nuclear hormone receptor superfamily and play essential roles in the physiological and pathological processes of cells [3]. PPARs have been demonstrated to have an important function in the endometrial trophoblast in studies [4, 5]. Therefore, PPAR pathway-related genes (PPAR-related gene) also participate in the occurrence and development of UCEC. Some PPAR ligands have an antiproliferative activity against endometrial cancer [6]. Inhibition of PPAR*γ* can promote the proliferation of endometrial cancer cells through the Bcl-2/caspase3 pathway [7].

Tumor somatic variation includes gene copy number alteration (CNA) and base mutation. Gene CNA includes amplification and deep deletion, which usually leads to changes in the related protein expression. Intentional mutations of genes include missense mutation, truncating mutation, splice mutation, and inframe mutation. Gene mutations can cause changes in protein amino acids, thereby affecting their standard structure and function. We found 83% of serous endometrial carcinoma had PPAR-related gene somatic variation, while 48% of endometrioid endometrial carcinoma had PPAR-related gene somatic variation. This variation frequency is quite large, and further exploration is needed.

In this study, we conducted a comprehensive analysis of the CNA, nucleotide mutation, and transcription statuses of the PPAR-related gene in UCEC. Furthermore, we discovered that a substantial number of PPAR-related genes are associated with patient prognosis. As a result, a *PPAR risk score* was developed to predict the prognosis of UCEC patients. It contributes to a better understanding of the PPAR pathway's function in UCEC and allows for a more precise management of UCEC patients after surgery.

## 2. Materials and Methods

### 2.1. PPAR Pathway-Related Gene Acquisition

Sixty-nine PPAR-related genes were obtained from a gene set (KEGG PPAR signaling pathway) in the molecular signature database (MSigDB) [8]. The systematic name of this pathway is M13088. The specific details of the 69 PPAR-related genes investigated in this study are listed in Supplementary Table S1.

### 2.2. Data Source and Study Population

A total of 539 putative copy number alteration data and 248 mutation data from whole-exome sequencing for endometrial cancer samples in the TCGA database [9] were downloaded from cBioPortal [10]. The R package ‘TCGAbiolinks' [11] was used to download the gene expression data and clinical data for 548 endometrial cancer samples in the TCGA-UCEC cohort. The Sankey plots used to display the samples' clinical information were drawn using the R package ‘ggalluvial' [12].

### 2.3. Pathway Enrichment Analysis

The differentially expressed genes between the PPAR-related gene CNA/mutation altered group and the unaltered group were determined by using the R package ‘edgeR.' Then, R package ‘clusterProfiler' [13] was ulilized to perform gene set enrichment analysis (GSEA). The hallmark gene sets (h.all.v7.2.symbols.gmt) were downloaded from the MSigDB.

### 2.4. Prognosis Analysis

The overall survival (OS) and disease-free survival (DFS) of each group of patients were calculated using Kaplan-Meier and log-rank analyses.

### 2.5. Immune Characteristic Analysis

We used the CIBERSORT [14] algorithm to calculate the infiltration status of 22 immune cells in the TCGA-UECE cohort and compared the results in each group of patients. The neoantigens data and of the TCGA-UCEC cohort are from previously published articles. The total tumor-infiltrating lymphocytes (TIL) regional fraction data and the neoantigens data of the TCGA-UCEC cohort were obtained from an authoritative article [15].

### 2.6. Prediction of Chemotherapy Response

The R package ‘pRRophetic' [16] and mRNA data were used to estimate each patient group's medication sensitivity. Among them, ridge regression was used to determine the samples' highest half-inhibitory concentration (IC50), and tenfold cross-validation was utilized to determine the accuracy.

### 2.7. PPAR Risk Score Generation

Through univariate and multivariate Cox regression analyses, the PPAR-associated gene most connected to patient prognosis was selected out. The linear combinational of the signature gene expression weighted by their regression coefficients was used to produce the *PPAR risk score* for each patient. The ‘pheatmap' R package was used to visualize the expression of each gene in *PPAR risk score*. The survival rate was calculated using the Kaplan-Meier method, and its statistical significance was determined using the log-rank test.

### 2.8. PPAR Risk Score Verification

The prediction model based on *PPAR risk score* was tested using univariate and multivariate cox regression analyses to see if it was an independent prognostic factor. The time-dependent receiver operating characteristic curve (TDROC) in the ‘survivalROC' [17] R package was used to examine *PPAR risk score*'s prediction ability at 1, 3, and 5 years.

### 2.9. Statistical Analyses

The clinical variables of different groups of patients were tested using Fisher's exact test or chi-square test. The Mann–Whitney *U* test or the Kruskal-Wallis test was utilized to compare the abundance of immune cell infiltration, neoantigens, and drug sensitivity between PPAR-related gene CNA/mutation altered and unaltered groups. *P* < 0.05 was considered statistically significant. The predictive nomogram was built with the R package ‘rms' and Iasonos' guide [18]. R (version 4.0.3) or GraphPad Prism 6.0 was used for all statistical tests and visual analysis (GraphPad Software, USA).

## 3. Results

### 3.1. The CNA Status of PPAR-Related Gene and Related Clinical Features in UCEC Patients

**Figure 1:**
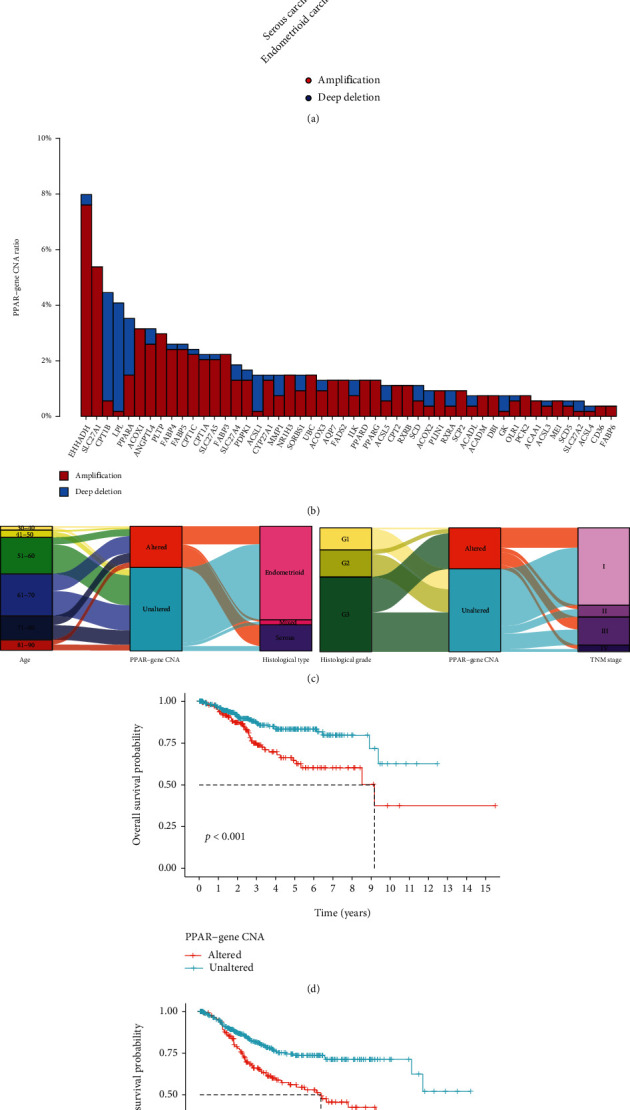
The CNA status of PPAR-related gene and related clinical features in UCEC patients. (a) The frequency of CNA alterations of PPAR-related gene in patients with different histological types. (b) The CNA type ratio of each PPAR-related gene. (c) Sankey plots show the clinical information of PPAR-related gene CNA and non-CNA patients. Kaplan-Meier curves show the correlation between PPAR-related gene CNA status and overall survival (d) or disease-free survival (e) probability of UCEC patients.

We analyzed the CNA status of each PPAR-related gene in UCEC. In general, PPAR-related gene copy number changes are observed in 80% of patients with serous endometrial carcinoma (Figures 1(a)), which is much more than that of patients with endometrioid endometrial carcinoma (less than 20%). The PPAR-related gene most prone to copy number amplification are EHHADH, SLC27A1, ACOX1, ANGPLT4, and PLTP (Figures 1(b)). The PPAR-related gene most prone to copy number deletion are CPT1B and LPL. However, PPARA has undergone a large amount of copy number amplification and deletion. As shown in Figures 1(c), there is no difference in the PPAR-related gene CNA status among all age groups and histological grades. However, CNA of PPAR-related gene occurs more in serous type and III-IV TNM stage patients. To evaluate the relationship between the PPAR-related gene CNA status and the prognosis of UCEC, we divided patients into the PPAR-related gene altered and unaltered groups and compared their survival probabilities. When PPAR-related gene CNA occurs, the probability of OS and DFS of patients was lower than that of CNA unaltered patients (Figures 1(d) and 1(e)).

### 3.2. The Mutation Status of PPAR-Related Gene and Related Clinical Features in UCEC Patients

**Figure 2:**
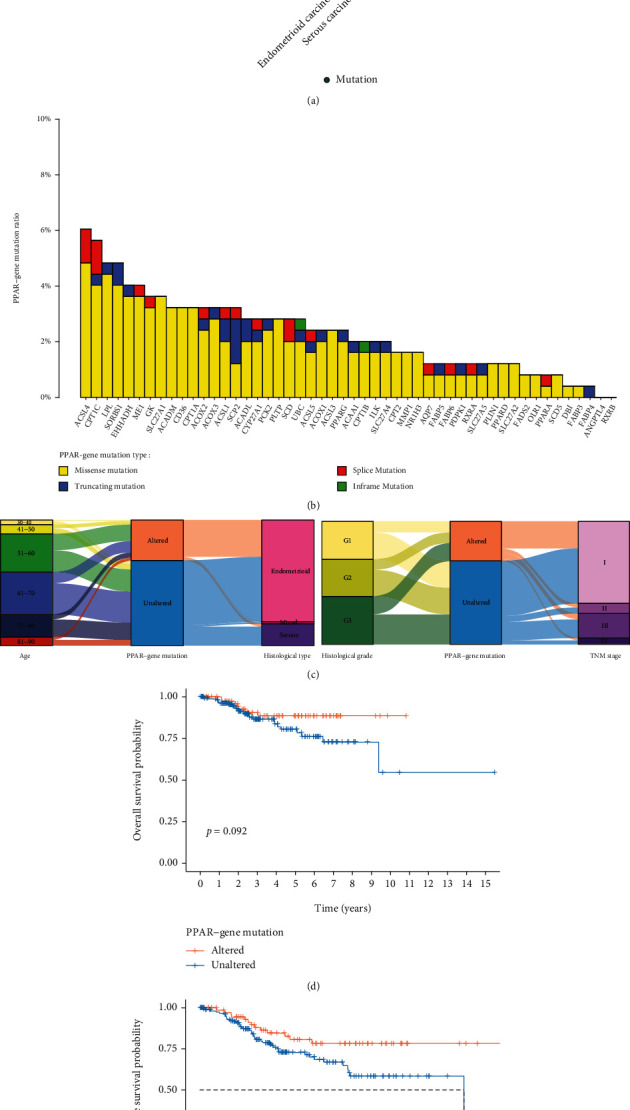
The mutation status of PPAR-related gene and related clinical features in UCEC patients. (a) The frequency of mutation alterations of PPAR-related gene in patients with different histological type. (b) The mutation type ratio of each PPAR-related gene. (c) Sankey plots shows the clinical information of PPAR-related gene mutated and nonmutated patients. Kaplan-Meier curves show the correlation between PPAR-related gene mutation status and overall survival (d) or disease-free survival (e) probability of UCEC patients.

In a similar way, we also analyzed the mutation status of each PPAR-related gene in UCEC. In general, the mutation of the PPAR-related gene is higher in endometrioid endometrial carcinoma than in serous endometrial carcinoma (Figure 2(a)). Commonly mutated PPAR-related genes are ACSL4, CPT1C, LPL, and SORBS1 (Figure 2(b)). A fascinating phenomenon is that EHHADH and LPL undergoes high copy number alterations and have high-frequency mutations in UCEC. As shown in Figures 2(c), there is no difference in the mutation probability of PPAR-related gene among all age groups and histological grades. Similarly, we divided patients into the PPAR-related gene mutated and nonmutated groups and compared their survival probabilities. Kaplan-Meier analysis of OS and DFS showed that the prognosis of patients with PPAR-related gene mutation was better than that of nonmutated patients (Figures 2(d) an(d) 2(e)).

### 3.3. Comparison of Transcriptomic Traits between PPAR-Related Gene Altered and Unaltered Patients

**Figure 3:**
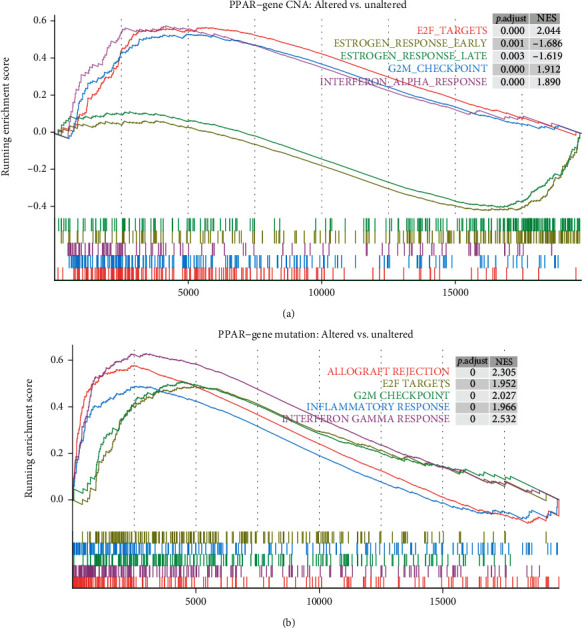
GSEA analysis between PPAR-related gene altered patients and unaltered patients. The five most significantly changed pathways in the PPAR-related gene CNA patients (a) and the PPAR-related gene mutation patients (b). *P*.adjust: adjusted *P* value; NES: normalized enrichment score.

To further analyze the potential biological changes in UCEC after PPAR-related gene CNA or mutation, we used the hallmark gene sets (h.all.v7.2.symbols.gmt) from the ‘MSigDB' to perform the GSEA between the PAPP-gene altered and nonaltered groups. The full 50 pathways/gene sets enriched with were presented in the Supplementary Materials—GSEA results. Based on the adjusted *P* value and normalized enrichment score, we selected the five pathways with the most significant changes for display. The results of GSEA showed that E2F TARGETS, G2M CHECKPOINT, and INTERFERON ALPHA RESPONSE pathways were significantly upregulated in the PPAR-related gene CNA altered group. In contrast, ESTROGEN RESPONSE pathways were significantly downregulated (Figure 3(a)). When PPAR-related gene mutations occur in UCEC patients, in addition to E2F TARGETS, G2M CHECKPOINT, and INTERFERON GAMMA RESPONSE pathways, the ALLOGRAFT REJECTION and INFLAMMATORY RESPONSE pathways were also significantly activated (Figure 3(b)).

### 3.4. Association between the PPAR-Related Gene Status and Tumor Immune Characteristics

**Figure 4:**
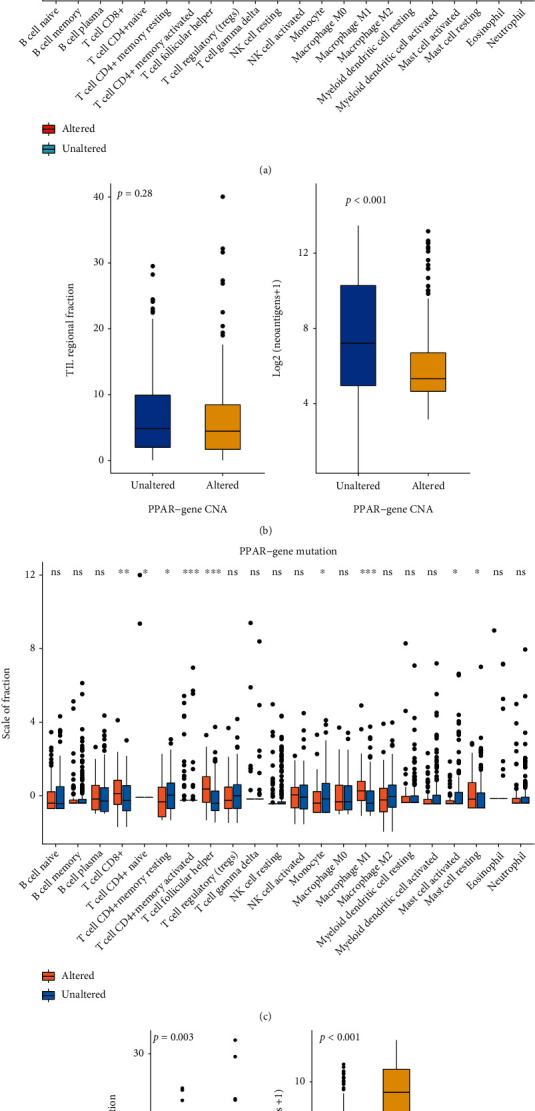
Tumor immune characteristics of patients with PPAR-related gene altered patients and unaltered patients. The relative abundance of tumor-infiltrating leukocytes (TILs) and neoantigens grouped by PPAR-related gene CNA status (a, b) and PPAR-related gene mutation status (c, d) in UCEC.

Through the analysis of the CIBERSORT algorithm, we found that the tumor microenvironment of UCEC patients with PPAR-related gene CNA has changed. Nevertheless, in UCEC patients with PPAR-related gene mutation, these changes were more prominent. In PPAR-related gene CNA patients, the enrichment of CD8^+^ T cell, Treg, and M1 type macrophage was reduced (Figure 4(a)). The total TIL fraction score did not change, but patients in the CNA group had fewer neoantigens (Figure 4(b)). In the PPAR-related gene mutation group, CD8+ T cell, T helper, and M1 type macrophage infiltration increased (Figure 4(c)). Also, in the PPAR-related gene mutation group, the total TIL immersion score increased, and more neoantigens (Figure 4(d)).

### 3.5. Prediction of Chemotherapy Therapy Outcomes in Patients with Different PPAR-Related Gene Status

UCEC patients use chemotherapy drugs for adjuvant treatment after surgery. In order to explore whether PPAR-related gene CNA and mutation status influence chemotherapy, we used the R package ‘pRRophetic' to evaluate the patient's (TCGA-UCEC cohort) sensitivity to the drugs. We selected four chemotherapy drugs commonly used in UCEC patients and predicted their IC50 for the PPAR-related gene alternated and nonaltered patients (Supplementary Figure S1). Cisplatin is a commonly used chemotherapy drug for patients with endometrial cancer. Through bioinformatics prediction, we found that patients with CNA and mutations of PPAR-related genes may be more sensitive to cisplatin (low IC50). Besides, we found that PPAR-related gene CNA patients were more sensitive to paclitaxel (*P* < 0.001) than unaltered patients but less sensitive to docetaxel (*P* < 0.001). There was no difference in sensitivity to doxorubicin between the two groups. We did not find any statistical difference in the above three drugs' sensitivity between patients with PPAR-related gene mutations and those without mutations.

### 3.6. The Transcription and Protein Expression of PPAR-Related Gene in UCEC Is Different from Normal Endometrium

**Figure 5:**
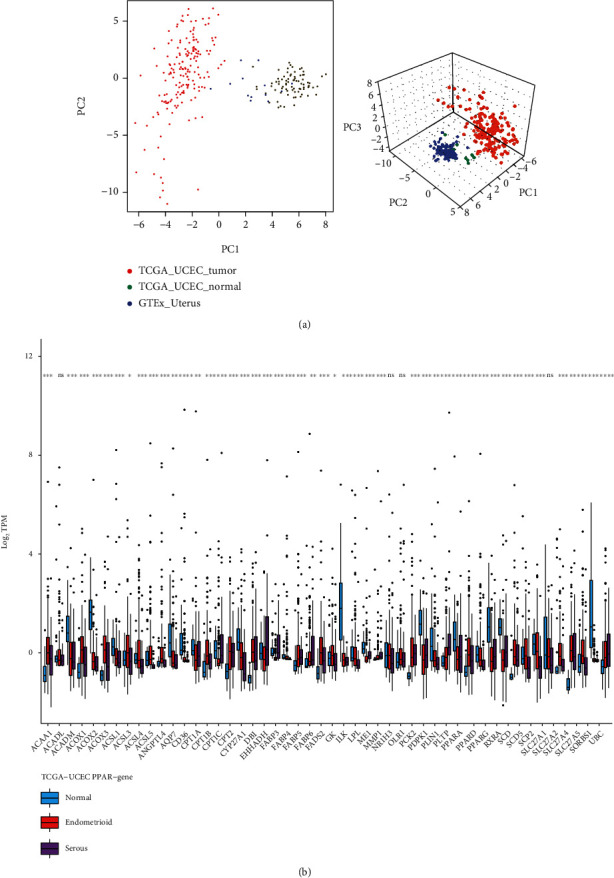
The transcription and protein expression analysis of PPAR-related-gene in UCEC. (a) The two-dimensional and three-dimensional PCA analysis results for PPAR-related-gene transcriptome. (b) The transcription level of each PPAR-related-gene between UCEC para-tumor tissues, endometrioid UCEC, and serous UCEC.

The CNA and mutation of genes ultimately perform biological functions by differentially changed RNA transcription and protein expression. We used RNA-seq and CPTAC (clinical proteomic tumor analysis consortium) protein expression data from UCEC patients and normal endometrium for further analysis. Firstly, we used principal component analysis (PCA) to describe the dimensionality reduction features of 50 PPAR-related genes. In the two-dimensional and three-dimensional PCA analysis results (Figure 5(a)), we found that PPAR-related gene can well distinguish UCEC (TCGA-UCEC-tumor), paratumor tissue (TCGA-UCEC-normal), and normal endometrium (GTEx-uterus). The results show that the expression of PPAR-related gene in these three tissues has different expression characteristics. We describe the transcription level of each gene in the PPAR-related gene set between UCEC paratumor tissues, endometrioid UCEC, and serous UCEC (Figure 5(b)). Similarly, we also analyzed the protein translation level of each PPAR-related gene (Supplementary Figure S2). The results showed that the RNA expression of EHHADH decreased in endometrioid UCEC and significantly increased in serous UCEC. But at the level of protein expression, we found that EHHADH was significantly increased in both types of UCEC. The LPL gene has high copy number deletions and missense mutations. Consistent with this, we have also observed a decrease in its transcriptome and proteome in UCEC. For other genes, some of them have the same trend in the transcriptome and proteome, but some are inconsistent.

### 3.7. The Transcription of PPAR-Related Gene Is Related to the Prognosis of UCEC Patients

**Figure 6:**
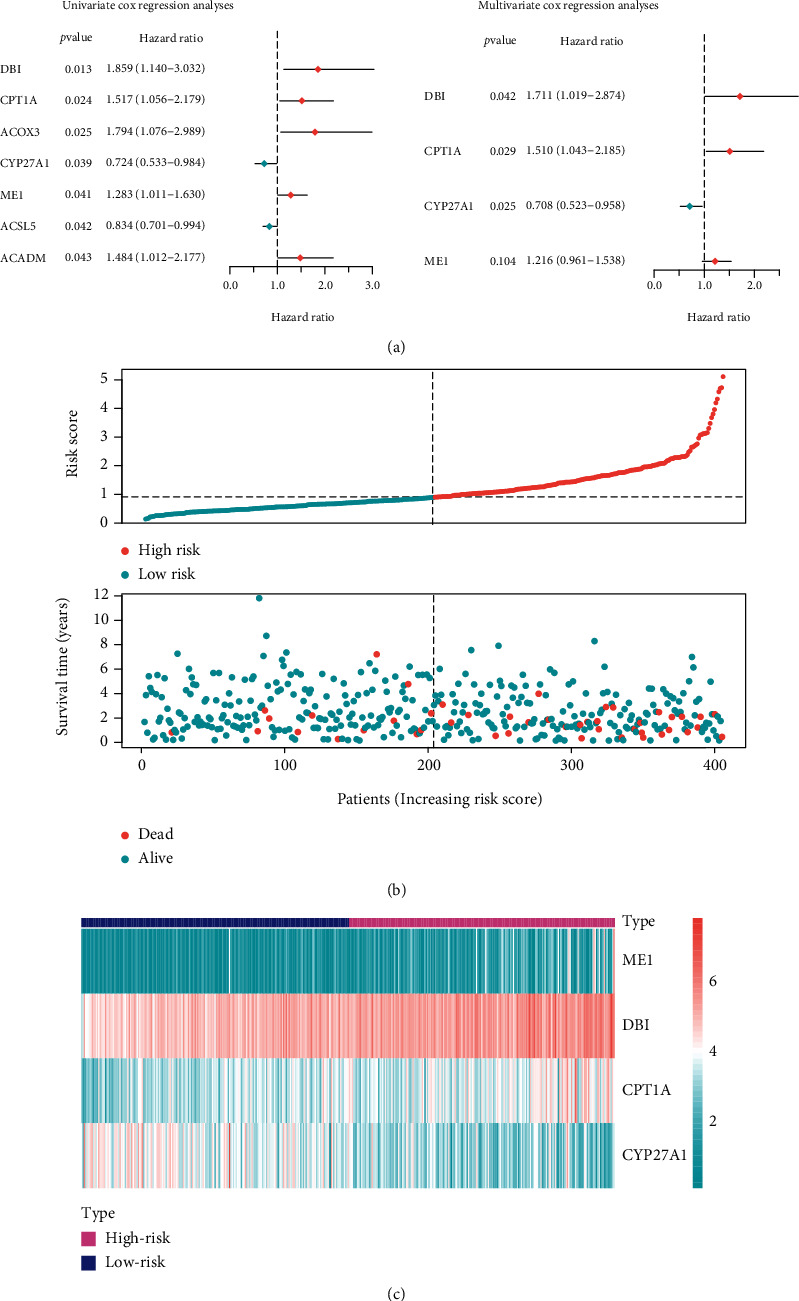
The construction of *PPAR risk score*. (a) Forest plot of the univariate and multivariate Cox regression analyses with the expression of PPAR-related gene. (b) The distribution of *PPAR risk score* and the survival status of patients with different scores. (c) Heatmap of the expression profiles of the *PPAR risk score* gene panel.

We performed univariate Cox regression analysis using TCGA-UCEC mRNA sequencing data and clinical data to investigate the relationship between the expression of PPAR-related genes and patient prognosis. As shown in Figure 6(a), we discovered seven genes that are substantially related to UCEC patient prognosis (*P* < 0.05, HR < 1 or HR > 1) and passed the proposed bootstrap test. For dimension reduction, the seven robust prognostic genes were subjected to multivariate Cox regression analysis. We discovered that the model composed of four genes functioned optimally (Figure 6(a)). Among them, CYP21A1 has a hazard ratio of less than one, implying that individuals who overexpress CYP21A1 live longer. Three genes (DBI, CPT1A, and ME1) with hazard ratios greater than one, on the other hand, have the opposite implication. We created a scoring system called *PPAR risk score* to predict the prognosis of UCEC patients based on the correlation coefficient of each gene.

(1) $$\begin{array}{c} \text{PPAR Risk score}=0.54*{\text{Exp}}_{\text{DBI}}+0.41*{\text{Exp}}_{\text{CPT}1\text{A}}-0.35*{\text{Exp}}_{\text{CYP}27\text{A}1}+0.20*{\text{Exp}}_{\text{ME}1}. \end{array}$$

We estimated the *PPAR risk score* for each UCEC patient. Patients were divided into two groups (high risk and low risk) according on their *PPAR risk score*, using the cohort's median as the cut-off value.  Figure 6(c) illustrates the distribution of *PPAR risk score* and patient survival status. The relative mRNA levels of such four genes between the two patient groups are depicted in Figure 6(d).

### 3.8. Independent Prognostic Value of the PPAR Risk score

**Figure 7:**
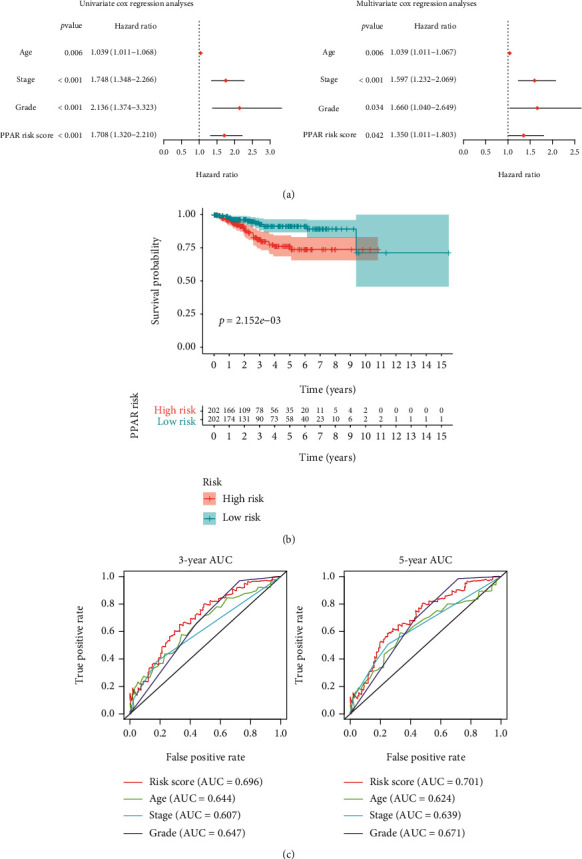
Verification of the *PPAR risk score's* independent prognostic value. (a) Forest plots of univariate and multivariate Cox regression analysis involving the *PPAR risk score* and clinical variables. (b) Overall survival Kaplan-Meier curves for patients in the high-risk and low-risk groups. (c) Curves of time-dependent receiver operating characteristic at one, three, and five years. AUC: area under curve.

The PPAR risk score is then compared to patient clinical data. The *PPAR risk score* was found to be a major independent risk factor for the overall survival rate of UCEC patients in both univariate and multivariate Cox regression analyses (Figure 7(a)). The Kaplan–Meier curve revealed that patients in the high-risk group had a significantly reduced survival rate (Figure 7(b)). The *PPAR risk score* outperformed the age, TMN stage, and pathological grade of UCEC patients in a one-year, three-year, and five-year ROC analysis (Figure 7(c)). These data imply that the *PPAR risk score* is a distinct prognostic factor that may be more effective in predicting patient outcome than existing clinical measures.

### 3.9. Develop a Prognostic Nomogram Based on PPAR Risk Score

**Figure 8:**
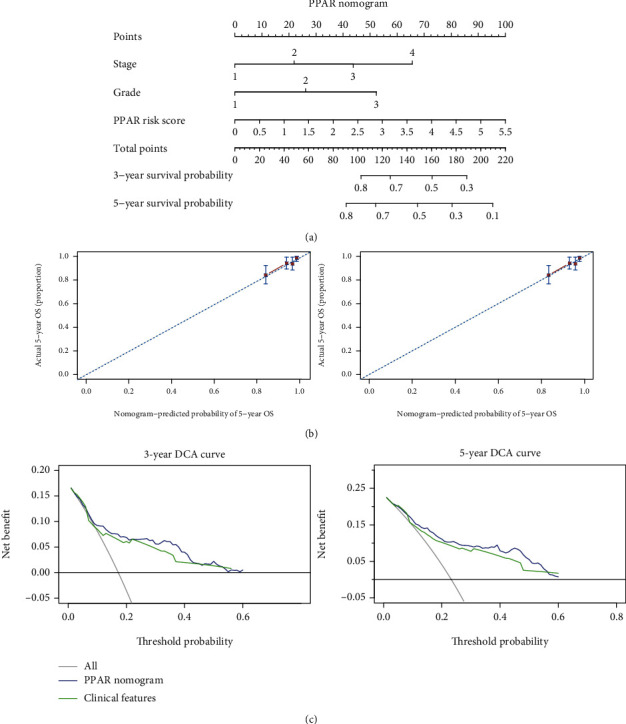
The establishment and verification of a nomogram based on the *PPAR risk score*. (a) A nomogram for estimating individual UCEC patients' 3- and 5-year survival chances. (b) Plots show how the nomogram was calibrated using the PPAR risk score in terms of consistency between anticipated and observed 3- and 5-year outcomes. (c) Nomogram decision curve studies for 3- and 5-year risks.

We developed a nomogram that integrates *PPAR risk score* and clinical prognostic factors to predict patients' 3- and 5-year survival rates (Figure 8(a)) in order to improve prognosis accuracy and ease clinical use. The patient's prognosis can be calculated using the sum of each factor's contribution scores.  Figure 8(b) shows that our nomogram outperforms an ideal model after three and five years of calibration. The clinical utility of our nomogram greatly outweighed the clinical features, according to the decision curve analysis (Figure 8(c)). It was discovered that using the *PPAR risk score* in combination with clinical features to predict prognosis could benefit more patients.

## 4. Discussion

Endometrial cancer is one of the primary gynecological malignancies globally. Its high-risk factors include disease stage, tumor size, grade, histological type, myometrial invasion, and lymph node metastasis [19]. It usually occurs in postmenopausal women, and the prognosis of late UCEC is very poor, which requires our focus. Like other cancers, the incidence and progression of UCEC also entail complicated molecular pathways [20]. Studies have demonstrated that the PPAR pathway plays a key role in UCEC [4–7]. Through bioinformatics research, we detected a substantial number of somatic mutations in the PPAR pathway-related genes in UCEC. Therefore, it is crucial to comprehensively examine the CNA and mutation status of PPAR pathway-related genes in UCEC.

This study found that a large proportion of PPAR-related gene CNAs were observed in patients with serous carcinoma. On the other hand, PPAR-related gene mutation frequency is higher in endometrioid endometrial cancer but not serous carcinoma. The PPAR-related gene most prone to CNA amplification is EHHADH, one of the four enzymes of the peroxisomal beta-oxidation pathway [21]. EHHADH can promote cisplatin resistance in bladder cancer cells [22]. Highly expressed EHHADH may play a similar function in UCEC, which requires more in-depth research.

To further analyze the biological changes in UCEC after PPAR-related gene CNA and mutation, we performed GSEA analysis. To further analyze the biological changes of UCEC when the PPAR-related gene somatic mutation occurs, we conducted pathway analysis. It can be concluded that when a somatic mutation of the PPAR-related gene occurs in UCEC, cell cycle-related pathways will be activated. Such as the E2F pathway and G2/M DNA damage checkpoint-related proteins. Gene expression in response to the interferon-gamma (IFN*γ*) pathway is significantly upregulated, common in other tumors [23]. Immune checkpoint blockade therapy can lead to upregulation of IFN*γ* and ultimately eliminate tumor cells. However, IFN*γ* signal can also induce tumor ischemia and homeostasis program, and the result is tumor clearance or tumor escape [24]. Therefore, the significant activation of IFN*γ* associated with PPAR-related gene somatic mutations in UCEC is a complicated research direction.

In the survival analysis, we found that PPAR gene CNA patients' survival time was significantly reduced compared with patients with unaltered CNA. On the other hand, compared with unaltered patients, PPAR-related gene mutation patients' survival time increased significantly. It is also an important conclusion we reached. It indicates that the somatic mutation status of PPAR-related gene may be an ideal prognostic predictor of UCEC. Clinicians can perform PPAR-related gene exon detection through the tumor tissue removed during a hysterectomy to predict the patient's prognosis and guide postoperative review and treatment plans.

The systemic treatment of advanced UCEC is usually chemotherapy and targeted therapy, but the outcome varies from person to person. The mRNA expression profile and the R software package ‘pRRophetic' were utilized to predict patients' six drug sensitivities and controls in this investigation. Cisplatin is helpful in individuals with PPAR-related gene somatic mutations, according to our findings. PPAR-related gene CNA patients have high sensitivity to paclitaxel but low sensitivity to docetaxel. These results may help clinicians choose chemotherapeutics for UCEC patients with PPAR-related gene somatic mutations.

In recent years, it has been shown that immune cells and inflammatory factors play a role in the tumor microenvironment. Sufficient activation of effector T cells are a prerequisite for the body to kill tumor cells [25]. The expression of programmed cell death-1 and programmed death ligand-1 are present in up to 80% of EC patients [26]. Immunotherapy has become a promising solution for the treatment of UCEC patients. T cells can recognize neoantigens (nonself-antigens) through HLA molecules on the surface of tumor cells. Many neoantigens provide opportunities for immunotherapy to trigger-specific and effective anticancer immune responses [27]. We found that the total TIL infiltration in the tumor microenvironment of patients with UCEC PPAR-related gene mutations increased, and more neoantigens were produced due to the mutations. It means that patients who have PPAR-related gene mutations may benefit from immunotherapy. It requires more clinical research results of UCEC immunotherapy to confirm, but it is also a good start.

The copy number alternation and mutation of genes ultimately influence cell biological functions by differentially changed RNA transcription and protein translation. In view of the great changes in the PPAR-related gene at the genome level, we further analyzed the RNA transcription and protein translation levels of the PPAR-related gene. Unsurprisingly, the RNA and protein expression levels of PPAR-related gene in UCEC are very different from normal endometrium. In some genes, we have observed consistent changes on these three levels, but in other genes, the changes are not one-to-one correspondence.

Because the expression of RNA can easily be measured from the patient's intraoperative pathological tissue, the RNA expression levels of certain genes in tumor section are new tools for predicting postoperative survival. PPAR-related gene expression varies greatly in different patients. We discovered that the PPAR-related gene panel is closely related to patients' postoperative survival time and can be used to predict patient prognosis. We discovered that CYP21A1, DBI, CPT1A, and ME1 are strongly connected to the prognosis of UCEC patients among the 50 PPAR-associated genes. Among them, CYP21A1 has a hazard ratio of less than one, implying that individuals who overexpress CYP21A1 live longer. The other three genes with hazard ratios greater than one, on the other hand, have the reverse implication. *PPAR risk score*, a prognostic prediction tool, was also developed. The *PPAR risk score* is a strong independent risk factor for the overall survival rate of UCEC patients, according to univariate and multivariate Cox regression analysis. We also developed a nomogram with *PPAR risk score* and clinical factors to make the findings of this study more practical in the clinic (Figure 8). The nomogram is a widely used method for predicting cancer prognosis. It combines the parameters of patients to predict their prognosis using statistical approaches. The accuracy of a nomogram is higher than that of a simple clinical profile of patients due to a combination of factors [18, 28]. The nomogram had better prediction accuracy and could benefit more patients, according to the calibration and decision curve analyses.

This research still has certain limitations. First, the study's initial data comes from cohort sequencing, and the findings must be confirmed by larger cohorts and molecular investigations. Second, in order to determine the appropriate cut-off value, the gene expression data used in this study must be revised. Third, because this is a retrospective study, the patient sample is heterogeneous, which could skew the findings. To confirm the utility of the *PPAR risk score* and nomogram established in this study, more clinical research is needed. In subsequent research, we will investigate and confirm the relation between the PPAR pathway and UCEC.

## 5. Conclusions

In conclusion, we found that PPAR-related gene somatic mutations often occur in UCEC patients. Patients with PPAR-related gene mutations may benefit from immunotherapy and cisplatin therapy. Furthermore, we found that the mRNA transcription level of PPAR-related gene in UCEC is significantly different from that of adjacent tissues/normal uterus. We constructed a scoring tool called *PPAR risk score* which is a strong independent risk factor for the overall survival rate of UCEC patients.

## Data Availability

The data used to support the findings of this study are available from the corresponding author upon request.

## Supplementary Materials

Supplementary 1 Supplementary Figure S1: prediction of chemotherapy treatment outcomes for patients with different PPAR-related gene statuses. Prediction of the sensitivity of four chemotherapy drugs in PPAR-related gene CNA patients (a) and mutation patients (b).

Supplementary 2 Supplementary Figure S2: the protein expression level of each PPAR-related-gene between UCEC para-tumor tissues, endometrioid UCEC and serous UCEC.

Supplementary 3 Supplementary Table S1: specific details about the PPAR-related gene investigated in this study.
