# Supplementary figures and images for: Comprehensive Analysis of Copy Number Variation, Nucleotide Mutation, and Transcription Level of PPAR Pathway-Related Genes in Endometrial Cancer
Source: PPAR Res. 2022 Jan 13;2022:5572258. doi: 10.1155/2022/5572258 (PMC8777464; doi:10.1155/2022/5572258)

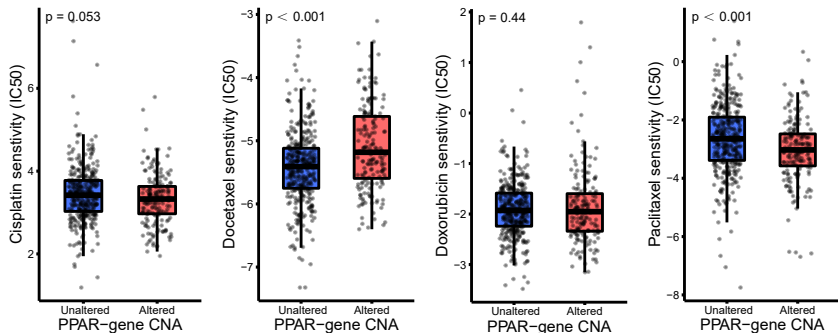

(a)

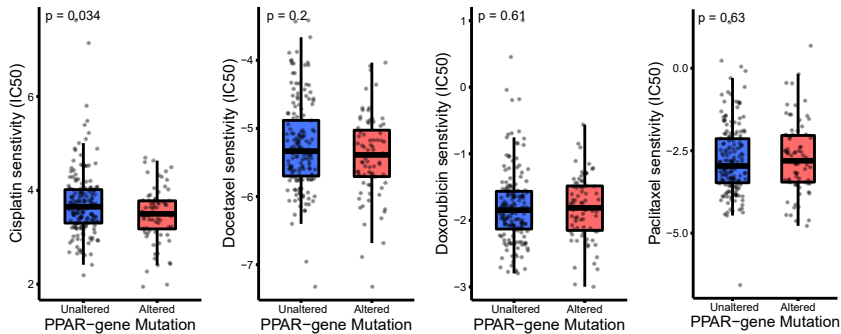

(b)

Supplement: Supplementary 1 — Supplementary Figure S1: prediction of chemotherapy treatment outcomes for patients with different PPAR-related gene statuses. Prediction of the sensitivity of four chemotherapy drugs in PPAR-related gene CNA patients (a) and mutation patients (b). [file 5572258.f1.pdf]

# CPTAC-UCEC PPAR-gene

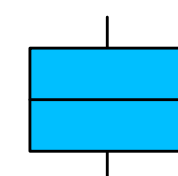

Normal

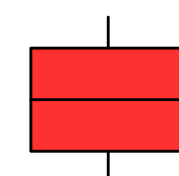

Endometrioid

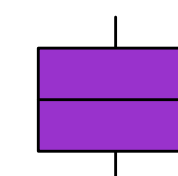

Serous

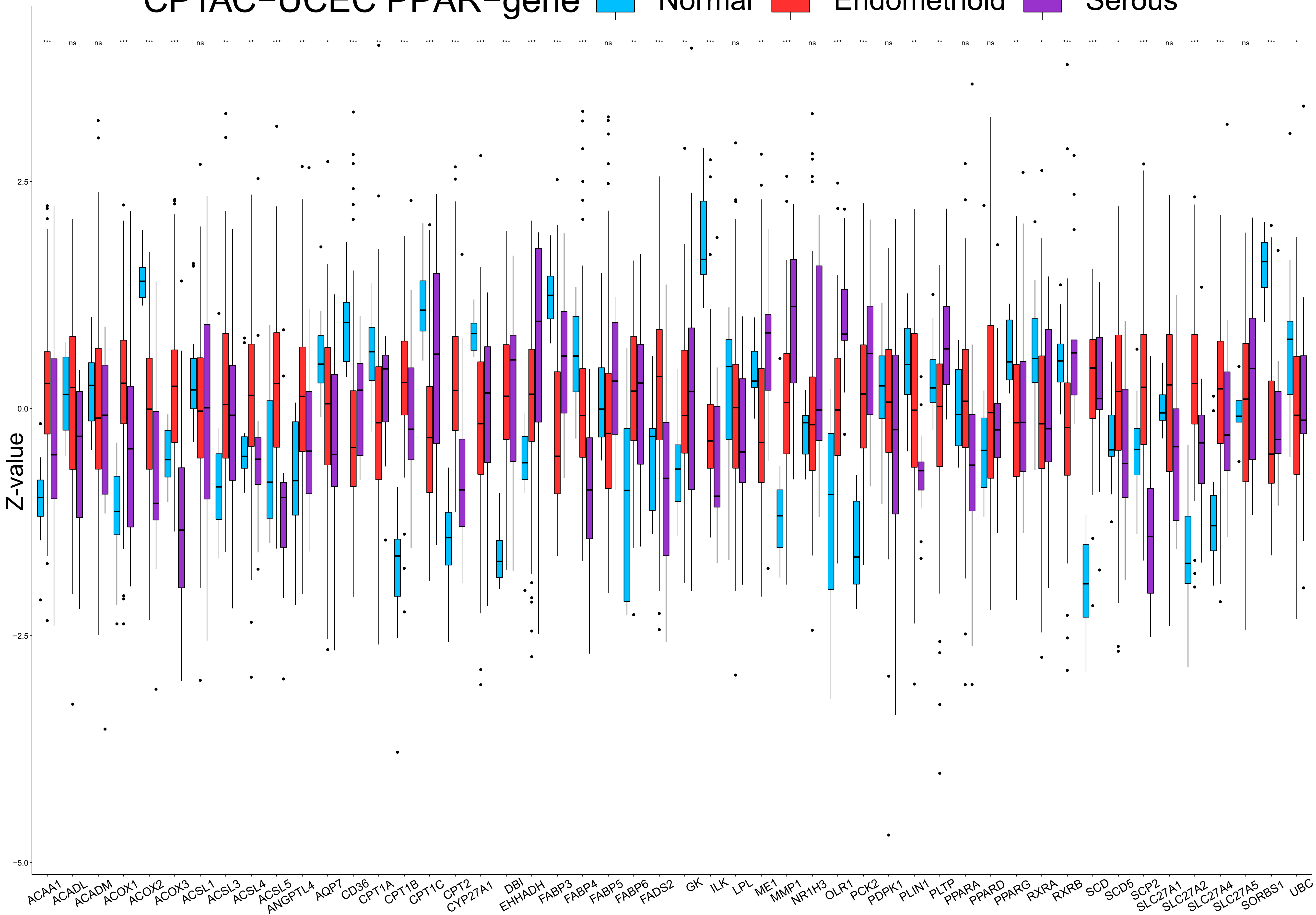

Supplement: Supplementary 2 — Supplementary Figure S2: the protein expression level of each PPAR-related-gene between UCEC para-tumor tissues, endometrioid UCEC and serous UCEC. [file 5572258.f2.pdf]
