# Supplementary material for: Comprehensive Analysis of Copy Number Variation, Nucleotide Mutation, and Transcription Level of PPAR Pathway-Related Genes in Endometrial Cancer
Source: PPAR Res. 2022 Jan 13;2022:5572258. doi: 10.1155/2022/5572258 (PMC8777464; doi:10.1155/2022/5572258)
Supplement: Supplementary 3 — Supplementary Table S1: specific details about the PPAR-related gene investigated in this study. [file 5572258.f3.docx]

**Supplementary Table S1. Specific details about the PPAR-related-gene investigated in this study.**

| **Entrez Gene ID** | **Gene Symbol** | **Description** |
| --- | --- | --- |
| 10062 | NR1H3 | NR1H3 - Oxysterols receptor LXR-alpha; Nuclear receptor. |
| 10580 | SORBS1 | SORBS1 - Sorbin and SH3 domain-containing protein 1; Plays a role in tyrosine phosphorylation of CBL by linking CBL to the insulin receptor. |
| 10998 | SLC27A5 | SLC27A5 - Bile acyl-CoA synthetase; Acyl-CoA synthetase involved in bile acid metabolism. |
| 10999 | SLC27A4 | SLC27A4 - Solute carrier family 27 (fatty acid transporter), member 1/4; Long-chain fatty acid transport protein 4; Involved in translocation of long-chain fatty acids (LFCA) across the plasma membrane. |
| 11001 | SLC27A2 | SLC27A2 - Very long-chain acyl-CoA synthetase; Acyl-CoA synthetase probably involved in bile acid metabolism. |
| 116519 | APOA5 | APOA5 - Apolipoprotein A-V; Minor apolipoprotein mainly associated with HDL and to a lesser extent with VLDL. |
| 126129 | CPT1C | CPT1C - Carnitine O-palmitoyltransferase 1, brain isoform; May play a role in lipid metabolic process; Belongs to the carnitine/choline acetyltransferase family. |
| 1374 | CPT1A | CPT1A - Carnitine O-palmitoyltransferase 1, liver isoform; Catalyzes the transfer of the acyl group of long-chain fatty acid-CoA conjugates onto carnitine, an essential step for the mitochondrial uptake of long-chain fatty acids and their subsequent beta-oxidation in the mitochondrion. |
| 1375 | CPT1B | CPT1B - Carnitine O-palmitoyltransferase 1, muscle isoform; Carnitine palmitoyltransferase 1B. |
| 1376 | CPT2 | CPT2 - Carnitine O-palmitoyltransferase 2, mitochondrial; Carnitine palmitoyltransferase 2. |
| 1579 | CYP4A11 | CYP4A11 - Cytochrome P450 4A11; Catalyzes the omega- and (omega-1)-hydroxylation of various fatty acids such as laurate, myristate and palmitate. |
| 1581 | CYP7A1 | CYP7A1 - Cholesterol 7-alpha-monooxygenase; Catalyzes a rate-limiting step in cholesterol catabolism and bile acid biosynthesis by introducing a hydrophilic moiety at position 7 of cholesterol. |
| 1582 | CYP8B1 | CYP8B1 - 7-alpha-hydroxycholest-4-en-3-one 12-alpha-hydroxylase; Involved in bile acid synthesis and is responsible for the conversion of 7 alpha-hydroxy-4-cholesten-3-one into 7 alpha, 12 alpha-dihydroxy-4-cholesten-3-one. |
| 1593 | CYP27A1 | CYP27A1 - Sterol 26-hydroxylase, mitochondrial; Catalyzes the first step in the oxidation of the side chain of sterol intermediates; the 27-hydroxylation of 5-beta- cholestane-3-alpha,7-alpha,12-alpha-triol. |
| 1622 | DBI | DBI - Diazepam-binding inhibitor (gaba receptor modulator, acyl-coa-binding protein); Acyl-CoA-binding protein; Binds medium- and long-chain acyl-CoA esters with very high affinity and may function as an intracellular carrier of acyl-CoA esters. |
| 1962 | EHHADH | EHHADH - Peroxisomal bifunctional enzyme; enoyl-CoA hydratase and 3-hydroxyacyl CoA dehydrogenase; In the C-terminal section; belongs to the 3- hydroxyacyl-CoA dehydrogenase family. |
| 2167 | FABP4 | FABP4 - Fatty acid-binding protein, adipocyte; Lipid transport protein in adipocytes. |
| 2168 | FABP1 | FABP1 - Fatty acid-binding protein, liver; Plays a role in lipoprotein-mediated cholesterol uptake in hepatocytes. |
| 2169 | FABP2 | FABP2 - Fatty acid-binding protein, intestinal; FABP are thought to play a role in the intracellular transport of long-chain fatty acids and their acyl-CoA esters. |
| 2170 | FABP3 | FABP3 - Fatty acid-binding protein, heart; FABP are thought to play a role in the intracellular transport of long-chain fatty acids and their acyl-CoA esters; Belongs to the calycin superfamily. |
| 2171 | FABP5 | FABP5 - Fatty acid-binding protein, epidermal; High specificity for fatty acids. |
| 2172 | FABP6 | FABP6 - Gastrotropin; Binds to bile acids and is involved in enterohepatic bile acid metabolism. |
| 2173 | FABP7 | FABP7 - Fatty acid-binding protein, brain; B-FABP could be involved in the transport of a so far unknown hydrophobic ligand with potential morphogenic activity during CNS development. |
| 2180 | ACSL1 | ACSL1 - Long-chain-fatty-acid--CoA ligase 1; Activation of long-chain fatty acids for both synthesis of cellular lipids, and degradation via beta-oxidation. |
| 2181 | ACSL3 | ACSL3 - Long-chain-fatty-acid--CoA ligase 3; Acyl-CoA synthetases (ACSL) activates long-chain fatty acids for both synthesis of cellular lipids, and degradation via beta-oxidation. |
| 2182 | ACSL4 | ACSL4 - Long-chain-fatty-acid--CoA ligase 4; Activation of long-chain fatty acids for both synthesis of cellular lipids, and degradation via beta-oxidation. |
| 23305 | ACSL6 | ACSL6 - Long-chain-fatty-acid--CoA ligase 6; Activation of long-chain fatty acids for both synthesis of cellular lipids, and degradation via beta-oxidation. |
| 2710 | GK | GK - Glycerol kinase; Key enzyme in the regulation of glycerol uptake and metabolism; Belongs to the FGGY kinase family. |
| 2712 | GK2 | GK2 - Glycerol kinase 2; Key enzyme in the regulation of glycerol uptake and metabolism; Belongs to the FGGY kinase family. |
| 284541 | CYP4A22 | CYP4A22 - Cytochrome P450 4A22; Catalyzes the omega- and (omega-1)-hydroxylation of various fatty acids such as laurate and palmitate. |
| 28965 | SLC27A6 | SLC27A6 - Long-chain fatty acid transport protein 6; Involved in translocation of long-chain fatty acids (LFCA) across the plasma membrane. |
| 30 | ACAA1 | ACAA1 - 3-ketoacyl-CoA thiolase, peroxisomal; acetyl-CoA acyltransferase 1. |
| 3158 | HMGCS2 | HMGCS2 - Hydroxymethylglutaryl-CoA synthase, mitochondrial; This enzyme condenses acetyl-CoA with acetoacetyl-CoA to form HMG-CoA, which is the substrate for HMG-CoA reductase. |
| 33 | ACADL | ACADL - Long-chain specific acyl-CoA dehydrogenase, mitochondrial; acyl-CoA dehydrogenase long chain; Belongs to the acyl-CoA dehydrogenase family. |
| 335 | APOA1 | APOA1 - Apolipoprotein A-I; Participates in the reverse transport of cholesterol from tissues to the liver for excretion by promoting cholesterol efflux from tissues and by acting as a cofactor for the lecithin cholesterol acyltransferase (LCAT). |
| 336 | APOA2 | APOA2 - Apolipoprotein A-II; May stabilize HDL (high density lipoprotein) structure by its association with lipids, and affect the HDL metabolism; Apolipoproteins. |
| 34 | ACADM | ACADM - Medium-chain specific acyl-CoA dehydrogenase, mitochondrial; Acyl-CoA dehydrogenase specific for acyl chain lengths of 4 to 16 that catalyzes the initial step of fatty acid beta- oxidation. |
| 345 | APOC3 | APOC3 - Apolipoprotein C-III; Component of triglyceride-rich very low density lipoproteins (VLDL) and high density lipoproteins (HDL) in plasma. |
| 3611 | ILK | ILK - Integrin-linked protein kinase; Receptor-proximal protein kinase regulating integrin- mediated signal transduction. |
| 364 | AQP7 | AQP7 - Aquaporin-7; Forms a channel for water and glycerol; Aquaporins. |
| 376497 | SLC27A1 | SLC27A1 - Solute carrier family 27 (fatty acid transporter), member 1/4; Long-chain fatty acid transport protein 1; Involved in translocation of long-chain fatty acids (LFCA) across the plasma membrane. |
| 4023 | LPL | LPL - Lipoprotein lipase; The primary function of this lipase is the hydrolysis of triglycerides of circulating chylomicrons and very low density lipoproteins (VLDL). |
| 4199 | ME1 | ME1 - Malate dehydrogenase (oxaloacetate-decarboxylating)(nadp+); Malic enzyme 1. |
| 4312 | MMP1 | MMP1 - Matrix metalloproteinase-1 (interstitial collagenase); Interstitial collagenase; Cleaves collagens of types I, II, and III at one site in the helical domain. |
| 4973 | OLR1 | OLR1 - Oxidized low-density lipoprotein receptor 1; Receptor that mediates the recognition, internalization and degradation of oxidatively modified low density lipoprotein (oxLDL) by vascular endothelial cells. |
| 51 | ACOX1 | ACOX1 - Peroxisomal acyl-coenzyme A oxidase 1; Catalyzes the desaturation of acyl-CoAs to 2-trans- enoyl-CoAs. |
| 5105 | PCK1 | PCK1 - Phosphoenolpyruvate carboxykinase, cytosolic [GTP]; Catalyzes the conversion of oxaloacetate (OAA) to phosphoenolpyruvate (PEP), the rate-limiting step in the metabolic pathway that produces glucose from lactate and other precursors derived from the citric acid cycle; Belongs to the phosphoenolpyruvate carboxykinase [GTP] family. |
| 5106 | PCK2 | PCK2 - Phosphoenolpyruvate carboxykinase [GTP], mitochondrial; Catalyzes the conversion of oxaloacetate (OAA) to phosphoenolpyruvate (PEP), the rate-limiting step in the metabolic pathway that produces glucose from lactate and other precursors derived from the citric acid cycle; Belongs to the phosphoenolpyruvate carboxykinase [GTP] family. |
| 51129 | ANGPTL4 | ANGPTL4 - Angiopoietin-related protein 4; Protein with hypoxia-induced expression in endothelial cells. |
| 5170 | PDPK1 | PDPK1 - 3-phosphoinositide-dependent protein kinase 1; Serine/threonine kinase which acts as a master kinase, phosphorylating and activating a subgroup of the AGC family of protein kinases. |
| 51703 | ACSL5 | ACSL5 - Long-chain-fatty-acid--CoA ligase 5; Acyl-CoA synthetases (ACSL) activate long-chain fatty acids for both synthesis of cellular lipids, and degradation via beta-oxidation. |
| 5346 | PLIN1 | PLIN1 - Perilipin-1; Modulator of adipocyte lipid metabolism. |
| 5360 | PLTP | PLTP - Phospholipid transfer protein; Facilitates the transfer of a spectrum of different lipid molecules, including diacylglycerol, phosphatidic acid, sphingomyelin, phosphatidylcholine, phosphatidylglycerol, cerebroside and phosphatidyl ethanolamine. |
| 5465 | PPARA | PPARA - Peroxisome proliferator-activated receptor alpha; Ligand-activated transcription factor. |
| 5467 | PPARD | PPARD - Peroxisome proliferator-activated receptor delta; Ligand-activated transcription factor. |
| 5468 | PPARG | PPARG - Peroxisome proliferator-activated receptor gamma; Nuclear receptor that binds peroxisome proliferators such as hypolipidemic drugs and fatty acids. |
| 6256 | RXRA | RXRA - Retinoic acid receptor RXR-alpha; Receptor for retinoic acid. |
| 6257 | RXRB | RXRB - Retinoic acid receptor RXR-beta; Receptor for retinoic acid. |
| 6258 | RXRG | RXRG - Retinoic acid receptor RXR-gamma; Receptor for retinoic acid. |
| 6319 | SCD | SCD - Stearoyl-coa desaturase (delta-9 desaturase); Acyl-CoA desaturase; Stearyl-CoA desaturase that utilizes O(2) and electrons from reduced cytochrome b5 to introduce the first double bond into saturated fatty acyl-CoA substrates. |
| 6342 | SCP2 | SCP2 - Non-specific lipid-transfer protein; Mediates in vitro the transfer of all common phospholipids, cholesterol and gangliosides between membranes. |
| 7316 | UBC | UBC - Polyubiquitin-C; Ubiquitin: Exists either covalently attached to another protein, or free (unanchored). |
| 7350 | UCP1 | UCP1 - Mitochondrial brown fat uncoupling protein 1; Mitochondrial protein responsible for thermogenic respiration, a specialized capacity of brown adipose tissue and beige fat that participates to non-shivering adaptive thermogenesis to temperature and diet variations and more generally to the regulation of energy balance (By similarity). |
| 79966 | SCD5 | SCD5 - Stearoyl-coa desaturase (delta-9 desaturase); Stearoyl-CoA desaturase 5; Stearyl-CoA desaturase that utilizes O(2) and electrons from reduced cytochrome b5 to introduce the first double bond into saturated fatty acyl-CoA substrates. |
| 8309 | ACOX2 | ACOX2 - 3alpha,7alpha,12alpha-trihydroxy-5beta-cholestanoyl-CoA 24-hydroxylase; Peroxisomal acyl-coenzyme A oxidase 2; Oxidizes the CoA esters of the bile acid intermediates di- and tri-hydroxycholestanoic acids; Belongs to the acyl-CoA oxidase family. |
| 8310 | ACOX3 | ACOX3 - Peroxisomal acyl-coenzyme A oxidase 3; Oxidizes the CoA-esters of 2-methyl-branched fatty acids; Belongs to the acyl-CoA oxidase family. |
| 9370 | ADIPOQ | ADIPOQ - Adiponectin, c1q and collagen domain containing; Adiponectin; Important adipokine involved in the control of fat metabolism and insulin sensitivity, with direct anti-diabetic, anti-atherogenic and anti-inflammatory activities. |
| 9415 | FADS2 | FADS2 - Acyl-coa 6-desaturase (delta-6 desaturase); Fatty acid desaturase 2; Component of a lipid metabolic pathway that catalyzes biosynthesis of highly unsaturated fatty acids (HUFA) from precursor essential polyunsaturated fatty acids (PUFA) linoleic acid (LA) (18:2n-6) and alpha-linolenic acid (ALA) (18:3n-3). |
| 948 | CD36 | CD36 - Platelet glycoprotein 4; Multifunctional glycoprotein that acts as receptor for a broad range of ligands. |
